# Triptolide ameliorates fine particulate matter-induced podocytes injury via regulating NF-κB signaling pathway

**DOI:** 10.1186/s12860-020-0248-6

**Published:** 2020-02-03

**Authors:** Qiang Wan, Zhongyong Liu, Ming Yang, Peng Deng, Nana Tang, Yanwei Liu

**Affiliations:** 1 Department of Medical Cardiology, The Affiliated Hospital of Jiangxi University of Traditional Chinese Medicine, Nanchang, 330006 China; 2 Key Laboratory of Modern Preparation of Traditional Chinese Medicine of Ministry of Education, Jiangxi University of Traditional Chinese Medicine, Nanchang, 330004 China

**Keywords:** Fine particulate matter, Podocytes, Triptolide, Nuclear factor kappa-B

## Abstract

**Background:**

PM2.5 is associated closely with an increased risk of membranous nephropathy (MN), however, whether PM2.5 could induce podocytes injury, the underlying pathology for MN, has not be thoroughly studied. Triptolide, an active component in *Tripterygium wilfordii* Hook F, is frequently used to treat MN in China, but its effects on PM2.5-induced podocytes injury is still largely unknown. Therefore, we evaluated the effects of PM2.5 on podocytes, and explored whether triptolide could improve PM2.5-induced podocytes injury and the possible underlying mechanisms.

**Results:**

Podocytes were incubated with PM2.5 after being pre-treated with triptolide, viability, apoptosis rate and migratory capacity of podocytes were determined by CCK-8 assay, flow cytometry and Transwell assay, respectively. Additionally, the levels of lactate dehydrogenase (LDH), malondialdehyde (MDA), and superoxide dismutase (SOD) in podocytes, the cytoskeleton of podocytes, the protein expressions of nephrin, podocin, Bcl-2, Bax, nuclear factor kappa-B/p65 (NF-κB/p65) and phospho-inhibitor of NF-κB (p-IκBα) were measured. Our data showed that PM2.5 treatment significantly increased the disorganization of F-actin stress fibers, the damaged structural integrity of nucleus, the deranged and dissociated cytoskeleton in podocytes, increased the podocytes apoptosis rate, the levels of MDA and LDH, markedly up-regulated the protein expression of Bax, NF-κB/p65 and p-IκBα, down-regulated the protein expression of nephrin, podocin and Bcl-2, and significantly decreased the level of SOD, the migration rate and the viability of podocytes, compared with those of the untreated podocytes. These effects of PM2.5 on podocytes, however, were reversed by triptolide administration.

**Conclusion:**

These results suggest that triptolide could prevent against PM2.5-induced podocytes injury via suppressing NF-κB signaling pathway.

## Background

Fine particulate matter with an aerodynamic diameter less than 2.5 μm (PM2.5), a complex mixture of liquid droplets and solid particles suspending in the atmosphere, exerts a significant adverse effect on human health. Published clinical and epidemiological studies have shown that short- and long-term exposure to PM2.5 increases mortality mainly due to respiratory and cardiovascular diseases [1–3]. Beside the direct effect on the lung, inhaled PM2.5 is able to translocate to other tissues causing impairment in humans. Recent studies have indicated that there is a link between exposure to PM2.5 and the increased rate of morbidity and mortality in patients with chronic kidney disease [4, 5]. Membranous nephropathy (MN), a leading cause of nephritic syndrome in adults, is a serious immune-mediated renal disease that is classified into primary MN and secondary MN related to various conditions, including systemic autoimmune disease, malignancy, infection and drug intoxication, can progress to end-stage renal disease [6]. Furthermore, data from an 11-year renal biopsy series including 71,151 patients from 938 hospitals spanning 282 cities in China show that long-term exposure to high levels of PM2.5 is associated closely with an increased risk of MN [7], however, the underlying mechanism of this association is poorly understood and has not been fully elucidated at cellular and molecular level.

MN is characterized by an accumulation of immune deposits on the outer aspect of the glomerular basement membrane (GBM) [8]. A pivotal pathogenesis of MN is podocytes (also called as glomerular visceral epithelial cells) injury either by creating an environment favorable to deposition and accumulation of immune complexes containing exogenous antigens or by providing a source of endogenous antigens. Once formed, these antigen-antibody complexes are capped and then attached to the GBM. The formation of subepithelial immune deposits, followed by complement activation, is responsible for the podocytes injury and proteinuria [9]. However, whether PM2.5 could induce podocytes injury is still largely unknown. Therefore, gaining a clearer understanding of podocytes injury induced by PM2.5 exposure is of vital significance in protecting public health, and reducing podocytes injury become important targets in the treatment of patients with MN. On the basis of these findings, we hypothesized that the inhalation of PM2.5 could accelerate the development of MN by inducing podocytes injury, and agents attenuating podocytes injury would be potential therapeutic drugs that prevent against PM2.5-induced MN.

Immunosuppressive therapy is widely used for MN, including corticosteroids, cyclophosphamide, chlorambucil, anti-proliferative agents (AP) such as azathioprine and mycophenolate mofetil, calcineurin inhibitors (CNI) such as tacrolimus and cyclosporine A [10]. However, these all predispose to opportunistic infections during long-term treatment, and severe adverse events are more frequent in patients given immunosuppressive therapy [10]. Thus, the use of immunosuppression is limited by the relative frequent occurrence of serious and potentially life threatening side effects. Triptolide, a diterpene triepoxide, one of the major active ingredients extracted from the medicinal plant *Tripterygium wilfordii* Hook F, exerts multiple biological activities in vivo and in vitro such as immune suppression, anti-inflammatory response and anti-tumor, is frequently used to treat autoimmune and/or inflammatory diseases such as systemic lupus erythematosus, rheumatoid arthritis, psoriasis and MN due to its favourable cost-benefit ratio [11–13]. Previous study showed that triptolide could markedly reduce proteinuria and podocytes injuries in MN rats without obvious adverse effects and protect against podocytes injury induced by the membrane attack complex of complement C5b-9 in vitro [14]. Although beneficial effects of triptolide on MN have been suggested, to date, the underlying mechanisms responsible for the amelioration of PM2.5-induced podocytes injury, have not been adequately studied.

Based on these pieces of evidence, we hypothesized that triptolide could prevent against PM2.5-induced MN by ameliorating podocytes injury. Therefore, we evaluated the effects of PM2.5 on podocytes in vitro, and then explored whether triptolide could improve PM2.5-induced podocytes injury and the possible underlying mechanisms in the current study.

## Results

### Source apportionment analysis

**Fig. 1:**
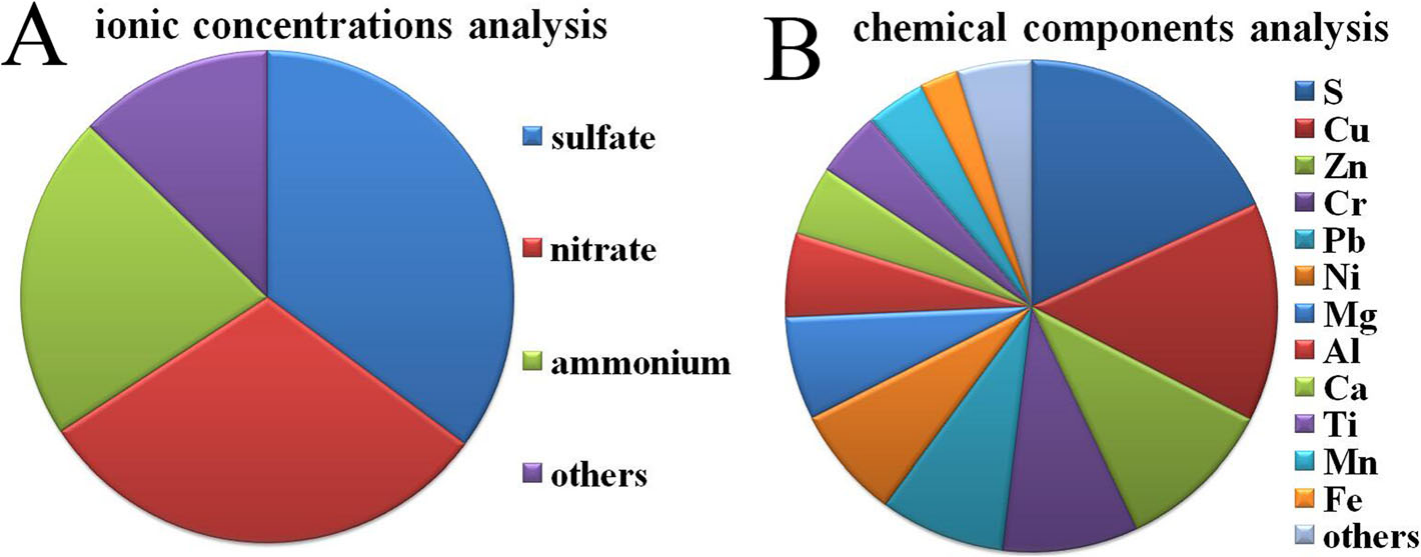
PM2.5 source apportionment analysis. Chemical components were detected based on inductively coupled plasma-atomic emission spectrometry and inductively coupled plasma mass spectrometry, respectively. **a** Ionic concentrations analysis. **b** Chemical components analysis. *n* = 3

The ionic concentrations analysis result (Fig. 1a) showed that sulfate, nitrate and ammonium had the highest contribution to the PM2.5 pollution in Nanchang. The chemical components analysis result (Fig. 1b) showed that S, Cu, Zn, Pb, Cr, Ni, Mg, Al, Ca, Ti, Mn and Fe were the major resources of PM2.5 pollution in Nanchang.

### Podocytes viability following PM2.5 or triptolide treatment

**Fig. 2:**
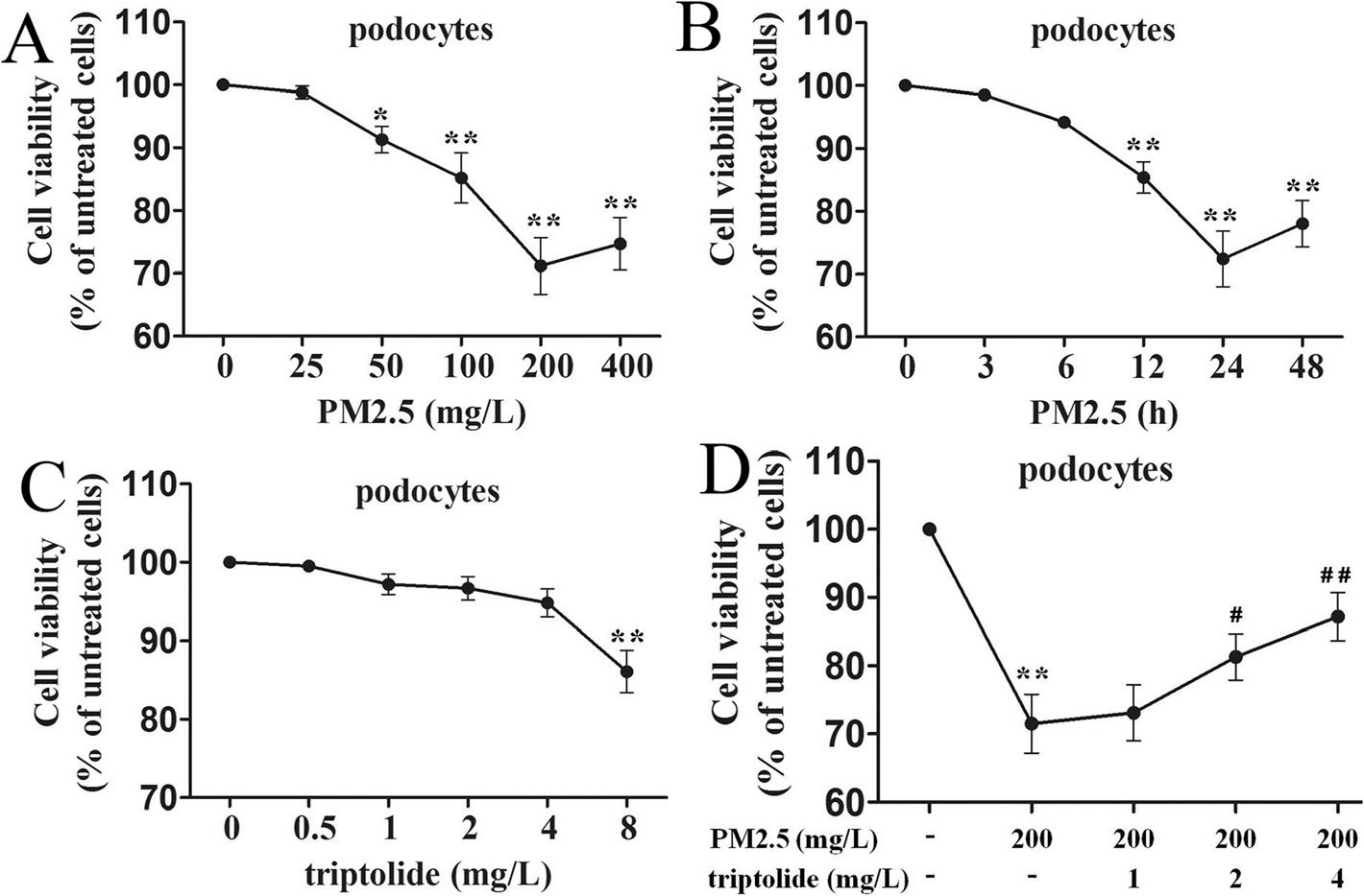
Podocytes viability following PM2.5 or triptolide treatment were assessed by CCK-8 assay. **a** Podocytes were treated with PM2.5 at 0, 25, 50, 100, 200 or 400 mg/L for 24 h. **b** Podocytes were treated with 200 mg/L PM2.5 for 0, 3, 6, 12, 24 or 48 h. **c** Podocytes were treated with triptolide at 0, 0.5, 1, 2, 4, 8 mg/L for 24 h. **d** Podocytes were pre-treated with triptolide at 0, 1, 2, 4 mg/L for 1 h and followed by the addition of 200 mg/L PM2.5 for 24 h. Data are expressed as mean ± SEM. *n* = 3. ^*^P < 0.05, ^**^P < 0.01 versus untreated cells; ^#^*P* < 0.05, ^##^*P* < 0.01 versus 200 mg/L PM2.5 group

In order to evaluate the podocytes injury induced by PM2.5, podocytes were stimulated with different concentrations of PM2.5 at 0, 25, 50, 100, 200 or 400 mg/L for 24 h, and podocytes were also stimulated with 200 mg/L PM2.5 for 0, 3, 6, 12, 24 or 48 h. Figure 2a demonstrated that podocytes viability markedly decreased at 50 mg/L, and a concentration of 200 mg/L PM2.5 lead to significant reduction in podocytes viability. Figure 2b indicated that podocytes viability markedly decreased at 12 h, and the treatment with 200 mg/L PM2.5 for 24 h lead to significant reduction in podocytes viability. Therefore, a treatment with 200 mg/L PM2.5 for 24 h was considered in subsequent experiments.

Subsequently, the cytotoxicity of triptolide on podocytes was examined by the CCK-8 assay. Podocytes were treated with triptolide at 0, 0.5, 1, 2, 4, 8 mg/L for 24 h. As shown in Fig. 2c, podocytes viabilities were not significantly affected by various concentrations of triptolide at 0 ~ 4 mg/L after a 24-h treatment, indicating that triptolide was non-toxic to podocytes below 4 mg/L. Thus, triptolide pre-treatment at concentrations of 0 ~ 4 mg/L were used for further experimentation.

Furthermore, we investigated the role of triptolide associated with PM2.5-induced podocytes viability reduction. As presented in Fig. 2d, the viability of PM2.5-treated podocytes was significantly decreased compared with that of the untreated cells, and the viabilities of 2, 4 mg/L triptolide-treated podocytes were significantly increased compared with that of the PM2.5-treated podocytes.

### Effect of triptolide on the apoptosis rate in the PM2.5-induced podocytes

**Fig. 3:**
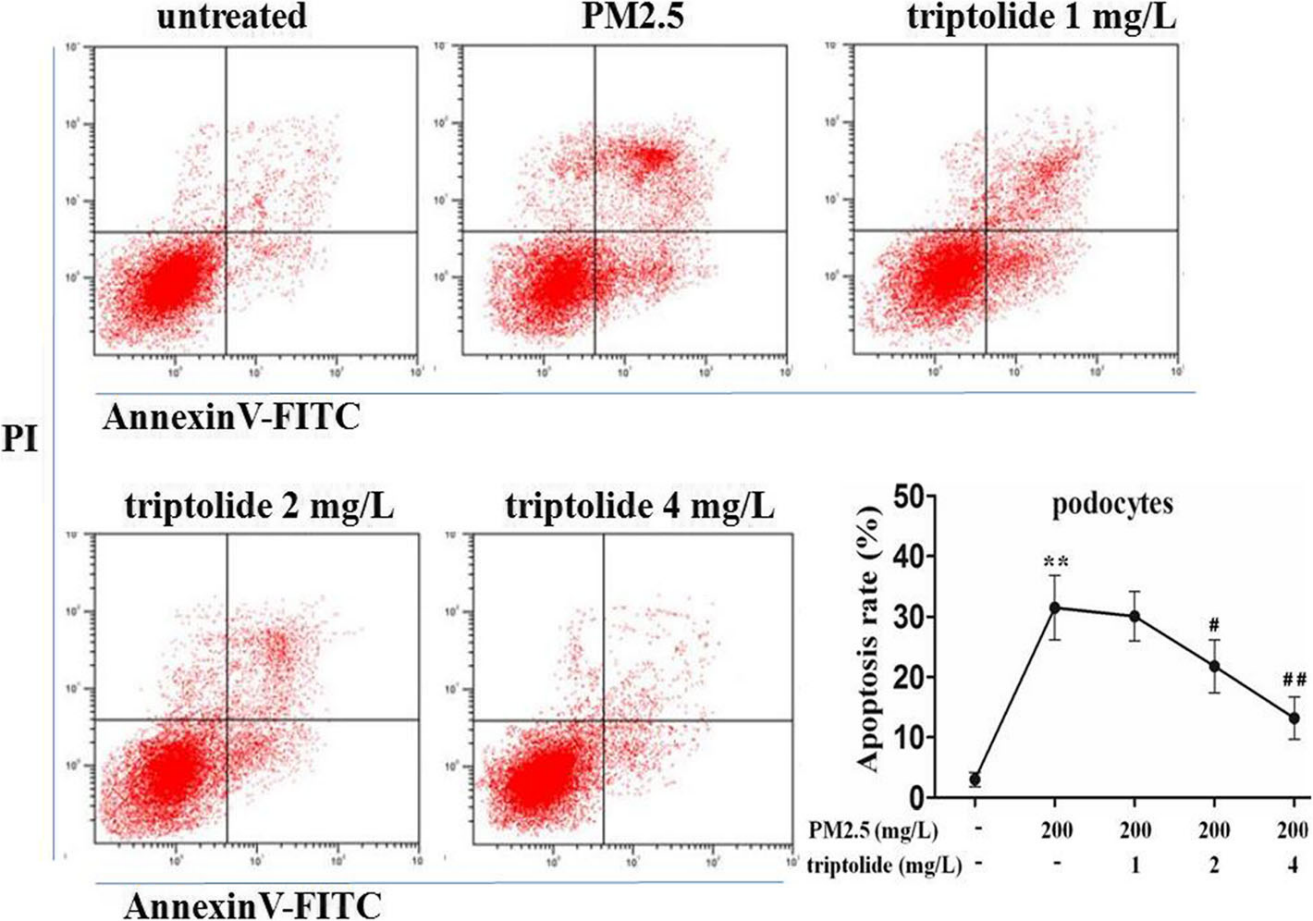
Apoptosis rates of podocytes were assessed by flow cytometry. Podocytes were pre-treated with triptolide at 0, 1, 2, 4 mg/L for 1 h and followed by the addition of 200 mg/L PM2.5 for 24 h. Data are expressed as mean ± SEM. *n* = 3. ^**^P < 0.01 versus untreated cells; ^#^*P* < 0.05, ^##^*P* < 0.01 versus PM2.5 group

The results of flow cytometry indicated that the apoptosis rate of the PM2.5-treated podocytes was significantly increased compared with that of the untreated cells, and the apoptosis rates of 2 or 4 mg/L triptolide-treated podocytes were significantly decreased compared with that of the PM2.5-treated podocytes (Fig. 3).

### Effect of triptolide on the migratory capacity in the PM2.5-induced podocytes

**Fig. 4:**
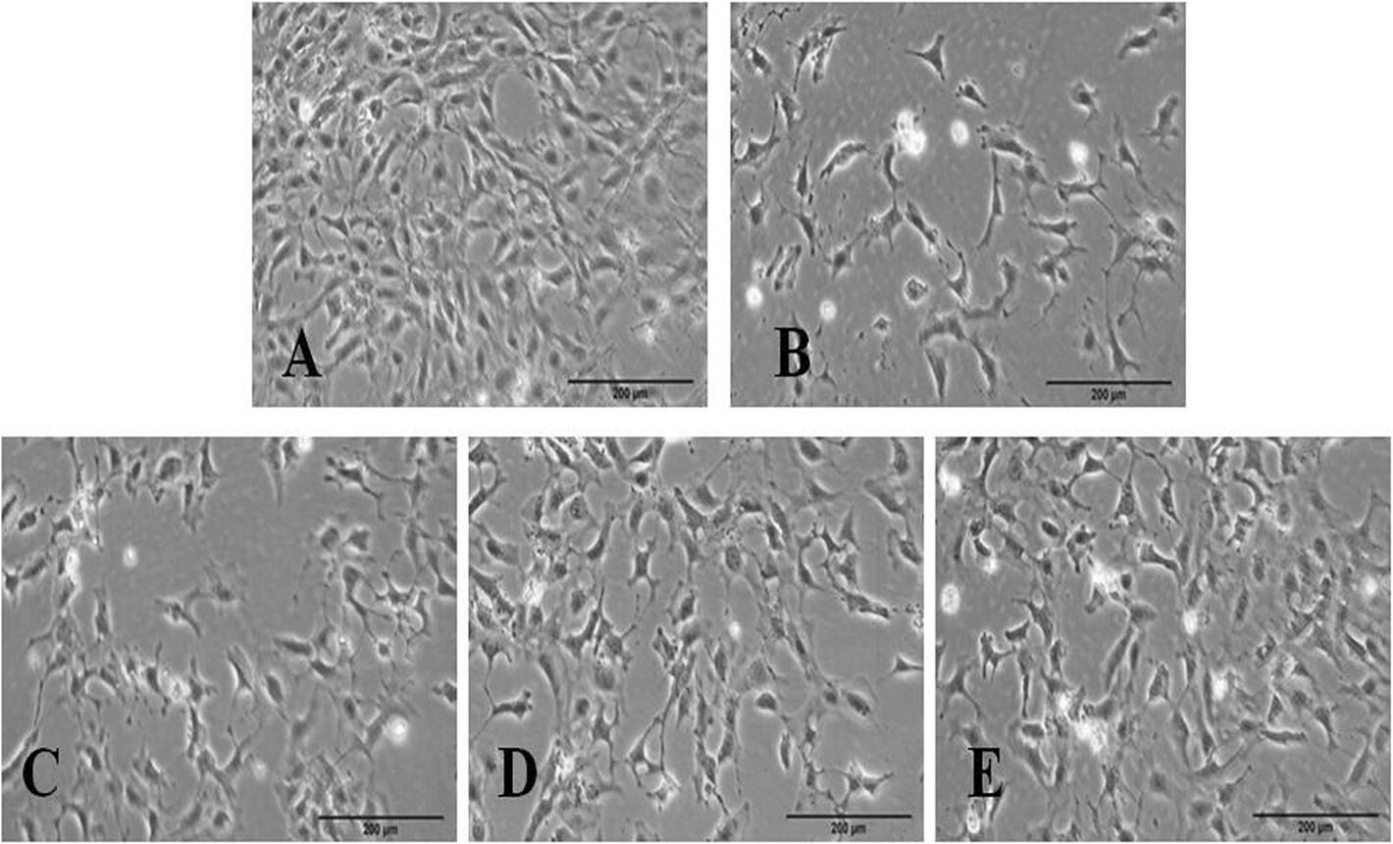
Migration of podocytes in each group. Representative Images of crystal violet-stained podocytes of the Transwell assay membranes (magnification × 200). **a** Untreated cells. **b** Podocytes were treated with 200 mg/L PM2.5 for 24 h. **c** Podocytes were pre-treated with 1 mg/L triptolide for 1 h and followed by the addition of 200 mg/L PM2.5 for 24 h. **d** Podocytes were pre-treated with 2 mg/L triptolide for 1 h and followed by the addition of 200 mg/L PM2.5 for 24 h. **e** Podocytes were pre-treated with 4 mg/L triptolide for 1 h and followed by the addition of 200 mg/L PM2.5 for 24 h. *n* = 3

The results of transwell migration assay demonstrated that the migration rate of the PM2.5-treated podocytes was significantly decreased compared with that of the untreated cells, and the migration rates of 2 or 4 mg/L triptolide-treated podocytes were significantly increased compared with that of the PM2.5-treated podocytes (Fig. 4).

### Effect of triptolide on the level of LDH in the PM2.5-induced podocytes

**Fig. 5:**
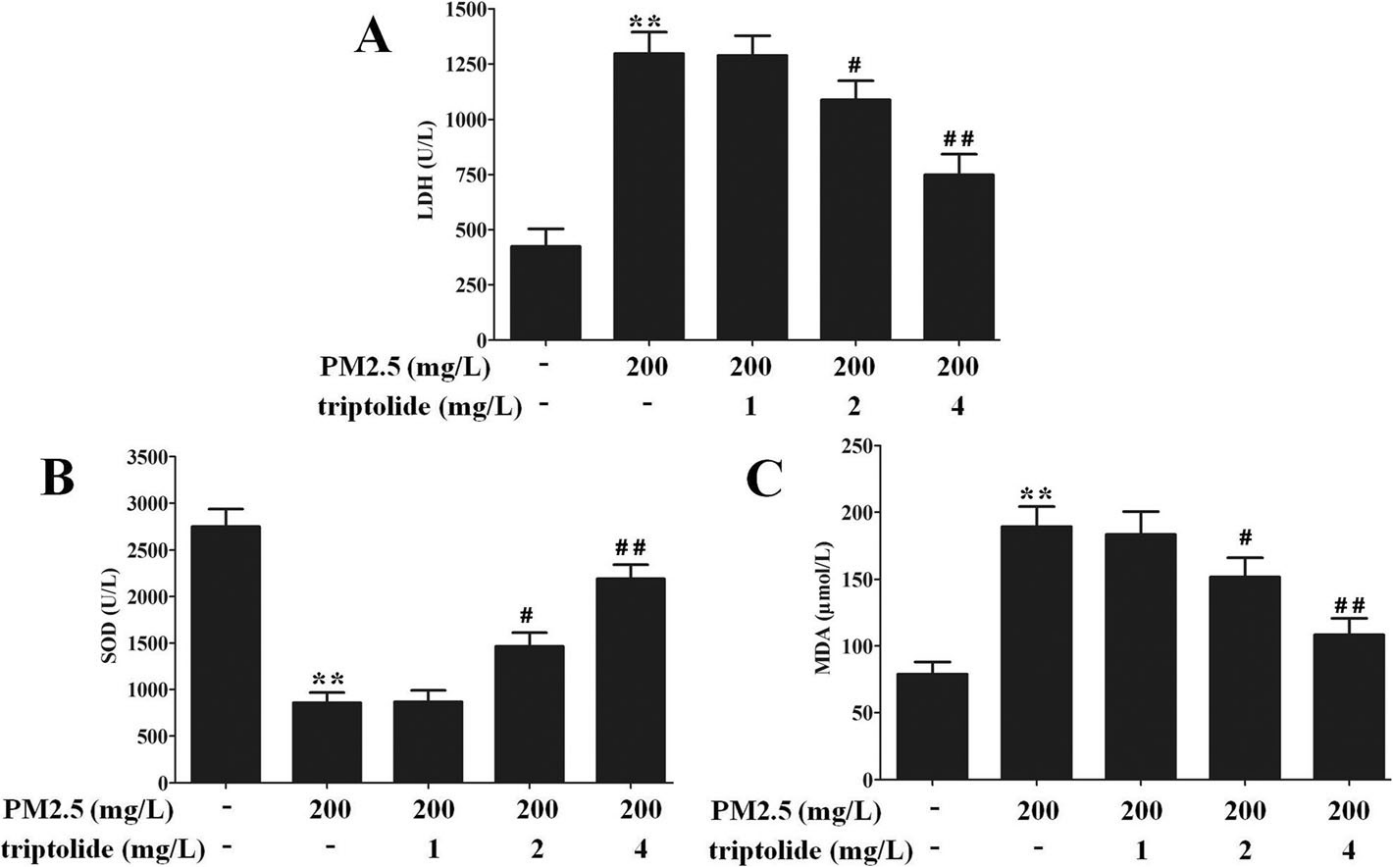
The levels of LDH, SOD and MDA in each group. **a** LDH level was measured by colorimetry. **b** SOD level was measured by colorimetric assay kit. **c** MDA level was measured by colorimetric assay kit. Data are expressed as mean ± SEM. *n* = 3. ^**^P < 0.01 versus untreated cells; ^#^*P* < 0.05, ^##^*P* < 0.01 versus 200 mg/L PM2.5 group

The level of LDH in the PM2.5-treated podocytes was significantly increased compared with that of the untreated cells, and the LDH levels of 2 or 4 mg/L triptolide-treated podocytes were significantly decreased compared with that of the PM2.5-treated podocytes (Fig. 5a).

### Effect of triptolide on the levels of SOD and MDA in the PM2.5-induced podocytes

Compared with the untreated cells, PM2.5 markedly increased the level of MDA, and decreased the level of SOD in podocytes. However, 2 or 4 mg/L triptolide administration significantly decreasced the level of MDA, and increased the level of SOD compared with the PM2.5-induced podocytes (Fig. 5b and c).

### Effect of triptolide on the cytoskeleton of the PM2.5-induced podocytes

**Fig. 6:**
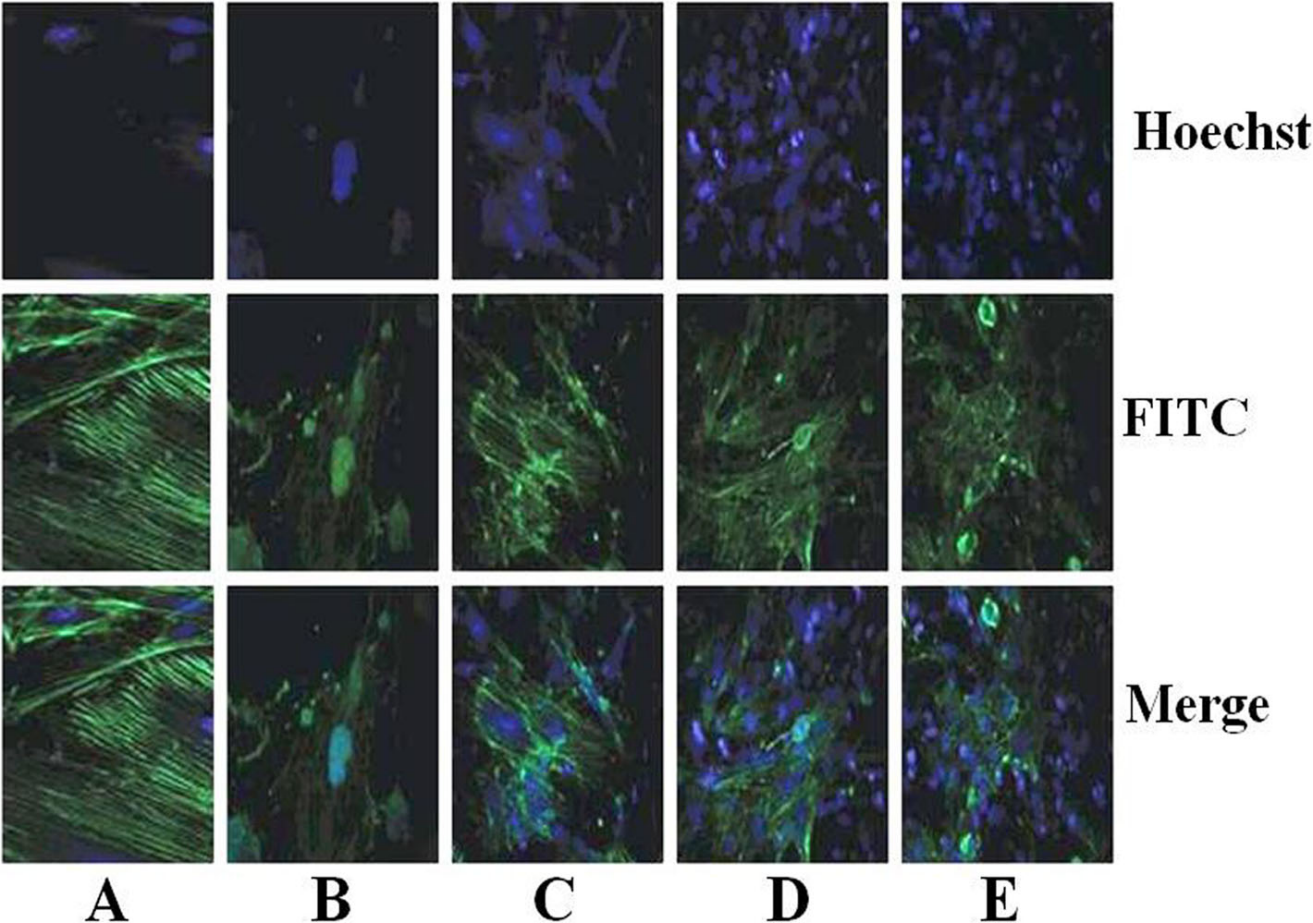
Cytoskeletal and nuclear staining of podocytes in each group (magnification × 400). **a** Untreated cells. **b** Podocytes were treated with 200 mg/L PM2.5 for 24 h. **c** Podocytes were pre-treated with 1 mg/L triptolide for 1 h and followed by the addition of 200 mg/L PM2.5 for 24 h. **d** Podocytes were pre-treated with 2 mg/L triptolide for 1 h and followed by the addition of 200 mg/L PM2.5 for 24 h. **e** Podocytes were pre-treated with 4 mg/L triptolide for 1 h and followed by the addition of 200 mg/L PM2.5 for 24 h. *n* = 3

As shown in Fig. 6, the skeleton of untreated podocytes arranged in parallel along the polarity, appeared bright green filaments with the microfilament structure being clear and intact. The nuclei were located in the center of podocytes, had regular shapes with blue staining. While, compared with the untreated podocytes, the microfilament structure of the PM2.5-treated podocytes was unclear and unintact, the reduced amount and disorganization of F-actin stress fibers were detected, the micronuclei and apoptotic bodies were predominantly observed, and nuclear areas and cytoplasm exhibited non-specifically stained. However, compared with the PM2.5-treated podocytes, the microfilament structures of 2 or 4 mg/L triptolide-treated podocytes were more clear and intact, increased amount of F-actin stress fibers, less micronuclei and apoptotic bodies were visible.

### Effect of triptolide on nephrin, podocin, Bcl-2, Bax, NF-κB/p65 and p-IκBα expressions in the PM2.5-induced podocytes

**Fig. 7:**
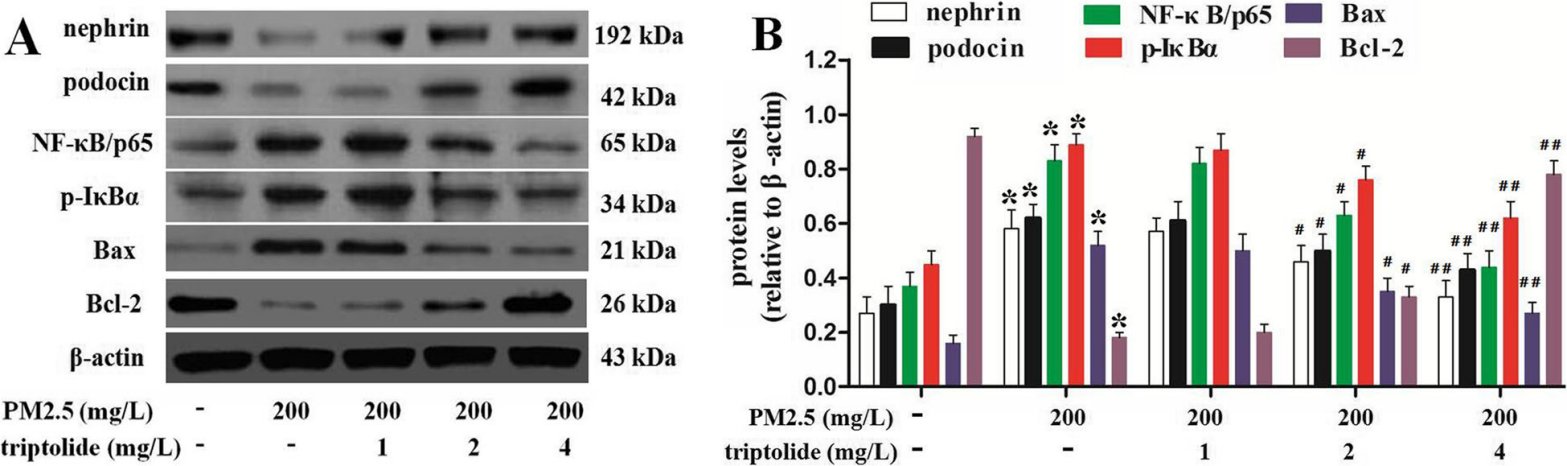
Nephrin, podocin, Bcl-2, Bax, NF-κB/p65 and p-IκBα protein expressions in podocytes were determined by Western blot. **a** Representative Western blot results for nephrin, podocin, NF-κB/p65, p-IκBα, Bax and Bcl-2 in podocytes. **b** Quantitative analysis of Western blot results. Data are expressed as mean ± SEM. *n* = 3. ^*^P < 0.01 versus untreated cells; ^#^*P* < 0.05, ^##^*P* < 0.01 versus 200 mg/L PM2.5 group

As shown in Fig. 7, PM2.5 markedly increased the protein expressions of Bax, NF-κB/p65 and p-IκBα, but significantly decreased the protein expressions of Bcl-2, nephrin and podocin in podocytes compared with those of the untreated cells. However, 2 or 4 mg/L triptolide administration significantly decreased the protein expressions of Bax, NF-κB/p65 and p-IκBα, but significantly increased the protein expressions of Bcl-2, nephrin and podocin in podocytes compared with those of the PM2.5-treated cells.

## Discussion

Nowadays, PM2.5 pollution has become one of the greatest urban issues in China. The residential population in Nanchang, the capital city of the Jiangxi province in eastern China, was 5.24 million and the number of vehicles reached 618,100 by the end of 2014 [15]. The chemical components analysis demonstrated that metallurgy sources and coal combustion (S, Zn, Cu and Pb), dust sources (Ca, Ti, Mg, Al, Fe and Mn) and vehicle exhaust (Ni and Cr) were the main resources of PM2.5 pollution in Nanchang. In addition, the ionic concentrations analysis indicated that the major source to the PM2.5 pollution in Nanchang was the combustion of fuel (such as residual oil and coal) due to the highest contribution of nitrate, ammonium and sulfate.

MN is a major cause of nephrotic syndrome of non-diabetic origin in adults. It is the leading glomerulopathy that recurs after kidney transplantation, and is the second or third leading cause of end-stage renal failure in patients with primary glomerulonephritis [16]. Higher level of PM2.5 exposure is associated with an increased risk of MN after adjusting for confounders including age, geographic region, gender, level of hospital for biopsy, clinical syndrome, pathologic laboratory, and year of biopsy [7]. Podocytes injury is an essential event involved in the pathogenesis of MN. Podocytes are highly specialized epithelial cells that cover the outer layer of the GBM. Podocytes serve as the final barrier to urinary protein loss through the special formation and maintenance of foot-processes and an interposed slit-diaphragm. Podocytes injury may cause podocyte detachment from the GBM, which leads to MN. F-actin is essential for the homeostasis of podocytes cytoskeleton, the change of F-actin may alter the cells phenotype, lead to foot process effacement, and induce podocytes injury [17]. In our current study, PM2.5 significantly increased the disorganization of F-actin stress fibers, the damaged structural integrity of nucleus, and the deranged and dissociated cytoskeleton of podocytes, indicated that PM2.5 may contribute to podocytes injury, and the PM2.5-induced podocytes injury model was successfully established.

Apoptosis of podocytes contributes to proteinuria in many chronic kidney diseases. As a pro-apoptotic protein, Bax expression induces hyperpolarization of mitochondria, cyt *c* release and mitochondrial network fragmentation. As an anti-apoptotic protein, Bcl-2 inhibits the stable integration of Bax into mitochondrial membranes hindering Bax activity [18]. Oxidative stress has been recognized as one of the most critical pathological factors involved in the evolution of MN, oxidative stress initiation induced by excess production of MDA and deficient production of SOD, and subsequent apoptosis have been thought to be associated with podocytes injury [19]. LDH is a soluble cytoplasmic enzyme that is present in almost all cells and is released into extracellular space when the plasma membrane is damaged [20]. LDH activity in the culture medium can, therefore, be used as an indicator of podocytes injury, and thus a measurement of PM2.5-induced cytotoxicity on podocytes. Nephrin, a slit diaphragm protein belonging to the immunoglobulin superfamily, is identified as a critical podocyte membrane component, maintaining the barrier function of the glomerular capillary wall [21]. Podocin, a membrane protein of the band-7-stomatin family, is considered to be localized on the membranes of podocyte pedicels, oligomerizes in lipid rafts together with nephrin to form the filtration slits [22]. Recently, it has been revealed that the decreased expression of both nephrin and podocin after podocyte injury, may contribute to the development of proteinuria in MN [23]. In the present study, PM2.5 treatment significantly increased the podocytes apoptosis rate, the levels of MDA and LDH, markedly up-regulated the protein expression of Bax and down-regulated the protein expression of nephrin, podocin and Bcl-2, but significantly decreased the level of SOD, the migration rate and the viability of podocytes, compared with those of the untreated podocytes, these findings strongly suggested that PM2.5-induced podocytes injury is related to oxidative stress.

Triptolide is a potent immunosuppressive, anti-inflammatory, anti-fertility and anti-tumor natural compound, and is widely used for the treatment of kidney diseases in China. Previous study have indicated that triptolide markedly ameliorated podocytes injury in a rat model of diabetic nephropathy, which may be due to the inhibition of macrophage infiltration in the kidney [24]. Mechanism research demonstrated that triptolide could protect against glomerular fibrosis and glomerular mesangial cells proliferation in rats by inhibition of the TGF-β1/Smad signaling pathway [25]. Furthermore, it has been reported that triptolide exerts novel protective effect on podocytes injury, triptolide effectively reduced the proteinuria induced by puromycin in nephrotic rats and the puromycin-induced podocytes injury through suppressing p38 mitogen-activated protein kinase activation [26]. In the present study, we performed in vitro cultures of podocytes and observed that PM2.5 induced significant podocytes injury. Fortunately, triptolide treatment decreased the cytoskeleton derangement and dissociation, restored the amount of F-actin stress fibers in the PM2.5-induced podocytes prominently. Additionally, triptolide treatment significantly decreased the apoptosis rate, the levels of MDA and LDH, markedly down-regulated the protein expression of Bax and up-regulated the protein expression of nephrin, podocin and Bcl-2, but significantly increased the level of SOD, the migration rate and the viability of the PM2.5-induced podocytes, these results demonstrated that triptolide protects podocytes from PM2.5-induced injury.

NF-κB is a nuclear transcription factor which regulates a series of transcription genes related to inflammation, oxidative stress and immunity, plays a critical role in the development of kidney diseases. Protein complex of NF-κB/p65 binds to IκBα and exists as an inactive form in the cytoplasm thereby blocking NF-κB nuclear translocation [27]. As an intracellular transcription factor system, NF-κB signaling pathway induced in response to various stimulations. Triptolide regulated renal tubular epithelial cells activity via inhibiting co-stimulatory molecule B7-H1 expression by decreasing NF-κB transcription [28]. Besides, recent study showed that triptolide treatment could significantly attenuate inflammatory response in a rat model of membranous glomerulonephritis by down-regulation of NF-κB signaling pathway [12]. To investigate whether the protective effect of triptolide on podocytes injury was associated with the regulation of NF-κB signaling pathway, western blot was performed to detect the protein expressions of NF-κB/p65 and p-IκBα in the PM2.5-induced podocytes in this experiment. The results indicated that PM2.5 markedly increased the protein expressions of NF-κB/p65 and p-IκBα compared with those of the untreated cells. Moreover, triptolide administration significantly decreased the protein expressions of NF-κB/p65 and p-IκBα compared with those of the PM2.5-treated cells. These observations suggested that podocytes injury could be induced by PM2.5 due to the activation of NF-κB signaling pathway, and triptolide could prevent against PM2.5-induced podocytes injury via suppressing NF-κB signaling pathway.

## Conclusion

In conclusion, our results demonstrated that triptolide could ameliorate PM2.5-induced podocytes injury, its protective effect is associated with suppressing NF-κB signaling pathway, leads us to find new approaches to target podocytes injury. This study provides insight for future studies on traditional Chinese medicine, however, the exact mechanism underlying this protective effect required further investigation.

## Methods

### Reagents

**Fig. 8:**
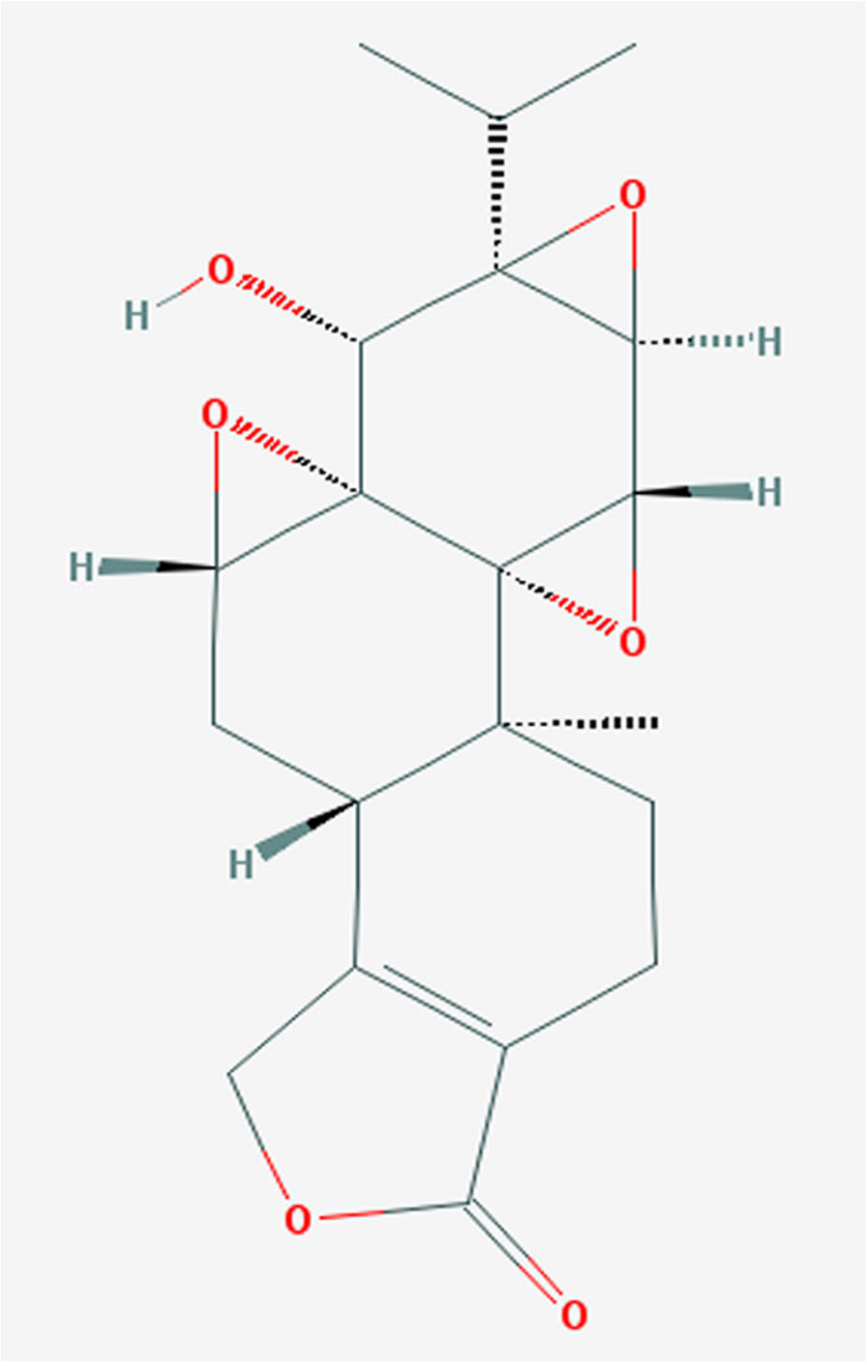
Chemical structure of triptolide

Triptolide (molecular formula, C_20_H_24_O_6_; chemical structure shown in Fig. 8) was obtained from National Institutes for Food and Drug Control (Beijing, China) and dissolved in 0.01% dimethyl sulfoxide (DMSO; Hyclone, Utah, USA). The purity (> 99%) of triptolide was detected by high-performance liquid chromatography. RPMI-1640 medium was purchased from Invitrogen (Carlsbad, CA, USA). Fetal bovine serum (FBS), penicillin and streptomycin were purchased from Gibco (Grand Island, NY, USA). Phosphatase and protease inhibitors, BCA protein assay kit were purchased from Applygen Technologies (Beijing, China). BSA and Hoechst 33342 were purchased from Sigma-Aldrich (St Louis, MO, USA). Recombinant murine interferon-훾 was purchased from Pepro Tech (Rocky Hill, NJ, USA). Lactate dehydrogenase (LDH) release kit was purchased from Promega (Madison, WI, USA). Malondialdehyde (MDA) and superoxide dismutase (SOD) were purchased from Nanjing Jiancheng Bioengineering Institute (Nanjing, China). Nephrin, podocin, Bcl-2, Bax, nuclear factor kappa-B (NF-κB)/p65, inhibitor of NF-κB (IκBα), phospho-IκBα (p-IκBα), β-actin and anti-mouse IgG antibodies were purchased from Cell Signaling Technology (Beverly, MA, USA).

### Collection and preparation of PM2.5

As previously described [29], PM2.5 samples were collected by using a low-volume sampler (24 L/min, continuous, non-concentrated, 24 h/day, 7 days/week, Beijing Geology Device Company, China) on the Bayi Road, a major artery around the centre of Nanchang, from January 1st to March 31th in 2018. After soaking in sterilize water for 30 min, the samples were facilitated with 20 min ultrasonic oscillation. The water-soluble components were extracted and kept at − 20 °C. PM2.5 was suspended and homogenized in DMEM for using.

### Source apportionment

As previously described [30], chemical components were calculated based on inductively coupled plasma-atomic emission spectrometry and inductively coupled plasma mass spectrometry. The cations and the anions contained in PM2.5 were estimated based on ion chromatography.

### Cell culture and treatment

Immortalized murine podocyte MPC-5 cell line [31] was kindly provided by Professor Dennis Murphy (Mount Sinai School of Medicine, Manhattan, USA). Podocytes were maintained in RPMI-1640 medium supplemented with 10% FBS, 100 U/mL penicillin and 100 μg/mL streptomycin. Podocytes were expanded by culture in a medium containing 10 U/mL recombinant murine interferon-훾 at 33 °C. Podocytes differentiation is induced by removal of interferon-훾 and thermo-shifting the cells from 33 °C to 37 °C for 7 d. Podocytes were starved from FBS for 24 h before all experiments. In the study, the podocytes injury model was established according to the reference [32] and the results of podocytes viability assay. To further elucidate the effect and the potential mechanism of triptolide on PM2.5-induced podocytes injury, the differentiated podocytes were incubated with 100 ng/mL PM2.5 for 24 h after being pre-treated with triptolide for 1 h.

### Determination of podocytes viability

Podocytes were seeded at a density of 1 × 10^4^ /well in 96-well plates and cultivated at 37 °C in a 5% CO_2_ incubator for 24 h. Then, medium was replaced with serum-free medium for another 24 h. Phosphate buffer saline (PBS) was added to cells as a control. After the above-mentioned pre-treatment, the medium was replaced with medium containing 10 μL CCK-8 for 2 h. Blank wells were set up that contained 10 μL CCK-8 only. Values of the absorbance (A) were detected at 540 nm using a Bio-Tek Plate Reader (BioTek Instruments, USA). Cell viability, which represents proliferation, was calculated using the following equation: Cell viability = [A (treatment) − A (blank)]/[A (PBS) − A (blank)].

### Determination of cell apoptosis

Podocytes were seeded in 6-well plates at a density of 1 × 10^5^ /well. After the above-mentioned pre-treatment, cells were harvested with 0.25% trypsin and washed with PBS. Cells were subjected to an apoptosis assay, using an Annexin V/FITC and PI apoptosis detection kit according to the manufacturer’s recommendation. Cell preparations were resuspended in 500 μL binding buffter, labeled with Annexin V/FITC and PI, and measured by a flow cytometer (BD Biosciences, NJ, USA).

### Podocytes migration assay

Capability of cell migration was examined by transwell migration assays. Podocytes were seeded on the upper chamber with serum-free medium at a density of 1 × 10^5^ /well, and RPMI-1640 medium containing 10% FBS as a chemoattractant was added to the lower chamber. Podocytes in upper chamber were discarded by using cotton wool after 24 h. Podocytes that migrated out of the upper chamber were fixed with 4% paraformaldehyde and stained with crystal violet. Subsequently, podocytes were captured by immunofluorescence microscope (Olympus, Tokyo, Japan) and counted by use of ImageJ software.

### Measurement of LDH release

The cytotoxic activity of PM2.5 on podocytes was evaluated in terms of LDH assay. After the above-mentioned pre-treatment, LDH release into the culture medium was measured by colorimetry using an enzyme detection kit according to the manufacturer’s instructions. The absorbance was measured spectrophotometrically at 490 nm. LDH level was extrapolated as the value detected in control cells, which was expressed as 1.

### Detection of oxidative stress biomarkers

After the above-mentioned pre-treatment, podocytes were washed with PBS and followed by radioimmunoprecipitation assay buffer, the lysates were analysed with the SOD and MDA kits following the manufacturer’s instructions, and the values of the absorbance were quantified at 570 nm to calculate the activity of SOD and MDA.

### Observation of the cytoskeleton

After the above-mentioned pre-treatment, podocytes were fixed with 4% paraformaldehyde, incubated with 0.1% Triton X-100 for 10 min and blocked with 5% FBS for 30 min in darkness, 10 μg/mL FITC-phalloidin was added to the central podocytes surface for 2 h, and Hoechst 33342 staining solution was then added at 37 °C in darkness for 10 min. Subsequently, anti-fade fluorescence mounting medium was added and the slides were detected and captured by immunofluorescence microscope (Olympus, Tokyo, Japan).

### Western blot analysis

Podocytes were lysed in cold RIPA buffer with phosphatase and protease inhibitors. The concentration of protein was quantified by BCA protein assay kit. Protein were separated by SDS-PAGE gels and transferred to polyvinylidene fluoride (PVDF) membranes (Millipore, USA). After blocked with 5% BSA, membranes were incubated separately with a primary 1:1000 dilution antibody (nephrin, podocin, Bcl-2, Bax, p65, p-p65, IκBα, p-IκBα and β-actin) at 4 °Covernight. After 3 washes with TBST, the membranes were further incubated with appropriate secondary antibody for 2 h at room temperature. Specific bands were detected using the ECL system and the electrophoresis image analyser (Bio-Rad, CA, USA), bands were normalized to β-actin expression.

### Statistical analysis

Experiments were performed at least three times. Data were analysed using SPSS 19.0 software and results were expressed as mean ± SEM. Statistical significance of the experimental data was analyzed by one-way analysis of variance (ANOVA), followed by Bonferroni post hoc test for multiple comparisons. *P* < 0.05 was considered statistically significant.

## Data Availability

The datasets used and/or analysed during the current study available from the corresponding author on reasonable request.

## Abbreviations

LDH: lactate dehydrogenase
MDA: malondialdehyde
MN: membranous nephropathy
NF-κB: nuclear factor kappa B
p-IκBα: phospho-inhibitor of NF-κB
PM2.5: fine particulate matter
SOD: superoxide dismutase
